# Supplementary material for: Case study of Argus in Togo: An SMS and web-based application to support public health surveillance, results from 2016 to 2019
Source: PLoS One. 2020 Dec 1;15(12):e0243131. doi: 10.1371/journal.pone.0243131 (PMC7707507; doi:10.1371/journal.pone.0243131)
Supplement: S1 Questionnaires — (PDF) [file pone.0243131.s001.pdf]

Supporting questionnaires

This supporting information is provided alongside the article: "Case study of Argus in Togo: an SMS and web-based application to support public health surveillance, results from 2016 to 2019".

Healthcare facility user-satisfaction questionnaire

1. Quel est votre **district sanitaire** ?	☐ District 1 ☐ District 2 ☐ District 3 ☐ District 4 ☐ District 5<br>☐ Cinkasse ☐ Kpendjal ☐ Tandjaore ☐ Tone ☐ Oti

2. Nom complet de la structure sanitaire	____

3.a) Avez-vous utilisé l'application Argus sur le téléphone pour envoyer des rapports hebdomadaires ou des alertes ?		☐ Oui ☐ Non
**Si OUI**	**De 1 (pire) à 5 (meilleur), merci de donner un score pour:**	
	3.b) l'apparence générale d'Argus sur le téléphone	☐ 1 ☐ 2 ☐ 3 ☐ 4 ☐ 5
	3.c) la documentation disponible pour utiliser Argus sur le téléphone	☐ 1 ☐ 2 ☐ 3 ☐ 4 ☐ 5
	3.d) la simplicité d'utilisation globale d'Argus sur le téléphone	☐ 1 ☐ 2 ☐ 3 ☐ 4 ☐ 5
	3.e) la simplicité d'envoi des rapports hebdomadaires avec Argus	☐ 1 ☐ 2 ☐ 3 ☐ 4 ☐ 5
	3.f) l'utilité d'Argus pour l'envoi des rapports hebdomadaires	☐ 1 ☐ 2 ☐ 3 ☐ 4 ☐ 5
	3.g) la simplicité d'envoi des alertes avec Argus	☐ 1 ☐ 2 ☐ 3 ☐ 4 ☐ 5
	3.h) l'utilité d'Argus pour l'envoi des alertes	☐ 1 ☐ 2 ☐ 3 ☐ 4 ☐ 5
	3.i) la simplicité de suivi du statut des précédents rapports et alertes envoyés sur Argus (historique)	☐ 1 ☐ 2 ☐ 3 ☐ 4 ☐ 5
	**Quels problèmes** avez-vous rencontrés depuis le début du pilote avec Argus sur le téléphone pour :	
	3.j) envoyer un rapport hebdomadaire ?<br>☐ Aucun problème<br>OU description du ou des problème(s) :<br><br>____	

	3.k) envoyer une alerte ?<br>☐ Aucun problème<br>OU description du ou des problème(s) :<br><br><br><br>____
	3.l) suivre le statut des précédents rapports et alertes envoyées (historique) ?<br>☐ Aucun problème<br>OU description du ou des problème(s) :<br><br><br><br>____
	3.m) Quelles **suggestions** pouvez-vous faire pour **améliorer Argus sur le téléphone** ?<br>☐ Aucune suggestion<br>OU suggestion(s) d'amélioration(s) :<br><br><br><br>____

4.a) Quel est le **diplôme de niveau le plus élevé** que vous avez obtenu ?	☐ BEPC collège ☐ BAC lycée ☐ Assistant d'hygiène d'État<br>☐ Technicien supérieur de santé ☐ Technicien supérieur de laboratoire<br>☐ Technicien supérieur de génie sanitaire ☐ Technicien biologiste<br>☐ Infirmier d'État ☐ Sage-femme d'État ☐ Médecin<br>☐ Autre, si autre précisez lequel :

4.a) Avez-vous un téléphone personnel mobile ?		☐ Oui ☐ Non
**Si OUI**	4.b) Depuis combien d'années ?	_ _ années

5.a) Avez-vous un téléphone personnel tactile (sans clavier) ?		☐ Oui ☐ Non
**Si OUI**	5.b) Depuis combien d'années ?	_ _ années

Merci

Intermediate and central-level user-satisfaction questionnaire

1. Dans quelle structure travaillez-vous ?<br>☐ DEPI ☐ DRS Lomé Commune ☐ DRS Savanes<br>☐ DPS District 1 ☐ DPS District 2 ☐ DPS District 3 ☐ DPS District 4 ☐ DPS District 5<br>☐ DPS Cinkasse ☐ DPS Kpendjal ☐ DPS Tandjaore ☐ DPS Tone ☐ DPS Oti

2.a) Avez-vous utilisé le site internet Argus pour valider les rapports hebdomadaires ou analyser leurs données?		☐ Oui ☐ Non
**Si OUI**	**De 1 (pire) à 5 (meilleur), merci de donner un score pour:**	
	2.b) l'apparence générale du site internet Argus	☐ 1 ☐ 2 ☐ 3 ☐ 4 ☐ 5
	2.c) la documentation disponible pour utiliser le site internet Argus	☐ 1 ☐ 2 ☐ 3 ☐ 4 ☐ 5
	2.d) la simplicité d'utilisation globale du site internet Argus	☐ 1 ☐ 2 ☐ 3 ☐ 4 ☐ 5
	2.e) la simplicité pour valider les rapports hebdomadaires sur le site internet Argus	☐ 1 ☐ 2 ☐ 3 ☐ 4 ☐ 5<br>☐ Ne sait pas
	2.f) l'utilité du site internet Argus pour valider les rapports hebdomadaire	☐ 1 ☐ 2 ☐ 3 ☐ 4 ☐ 5<br>☐ Ne sait pas
	2.g) la simplicité pour voir et télécharger les résumés de situation épidémiologique hebdomadaire sur le site internet Argus	☐ 1 ☐ 2 ☐ 3 ☐ 4 ☐ 5<br>☐ Ne sait pas
	2.h) la simplicité pour voir les rapports de promptitude/complétude ainsi que l'évolution du nombre de cas sur le site internet Argus	☐ 1 ☐ 2 ☐ 3 ☐ 4 ☐ 5<br>☐ Ne sait pas
	2.i) l'utilité du site internet Argus pour l'analyse des données	☐ 1 ☐ 2 ☐ 3 ☐ 4 ☐ 5<br>☐ Ne sait pas
	**Quels problèmes** avez-vous rencontrés depuis le début du pilote avec le site internet Argus pour :	
	2.j) valider les rapports hebdomadaires ?<br>☐ Aucun problème<br>OU description du ou des problème(s) :<br><br>____	

	3.k) voir et télécharger les résumés de situation épidémiologique hebdomadaire ou voir les rapports de promptitude/complétude ainsi que l'évolution du nombre de cas?<br>☐ Aucun problème<br>OU description du ou des problème(s) :<br><br><br><br><br>____
	3.m) Quelles **suggestions** pouvez-vous faire pour **améliorer le site internet Argus**?<br>☐ Aucune suggestion<br>OU suggestion(s) d'amélioration(s) :<br><br><br><br><br>____

**En général, à quelle fréquence** utilisez-vous le site internet Argus pour :
2.j) valider les rapports hebdomadaires ?<br>☐ une fois par semaine ☐ une fois toutes les deux semaines ☐ une fois par mois<br>☐ une fois par trimestre ☐ une fois par semestre ☐ une fois par an ☐ autre, si autre préciser :<br>☐ Jamais
3.k) pour visualiser et analyser les données ?<br>☐ une fois par semaine ☐ une fois toutes les deux semaines ☐ une fois par mois<br>☐ une fois par trimestre ☐ une fois par semestre ☐ une fois par an ☐ autre, si autre préciser :<br>☐ Jamais
3.m) pour identifier des évènements de santé publique ?<br>☐ une fois par semaine ☐ une fois toutes les deux semaines ☐ une fois par mois<br>☐ une fois par trimestre ☐ une fois par semestre ☐ une fois par an ☐ autre, si autre préciser :<br>☐ Jamais

4.a) Des investigations ont-elles été déclenchées à partir des données recueillies par Argus (rapports hebdomadaires et alertes) ?		☐ Oui ☐ Non ☐ Ne sait pas
**Si OUI**	4.b) Combien depuis le début du pilote	_ _ _

4.a) Utilisez-vous le résumé de situation épidémiologique hebdomadaire pendant les réunions d'équipe ?	☐ Oui ☐ Non ☐ Ne sait pas

4.a) Quel est le **diplôme de niveau le plus élevé** que vous avez obtenu ?	☐ BEPC collège ☐ BAC lycée ☐ Assistant d'hygiène d'État<br>☐ Technicien supérieur de santé ☐ Technicien supérieur de laboratoire<br>☐ Technicien supérieur de génie sanitaire ☐ Technicien biologiste<br>☐ Infirmier d'État ☐ Sage-femme d'État ☐ Médecin<br>☐ Autre, si autre précisez lequel :

4.a) Avez-vous un ordinateur personnel ?		☐ Oui ☐ Non
**Si OUI**	4.b) Depuis combien d'années ?	_ _ années

5.a) Allez-vous sur internet pour votre usage personnel ?		☐ Oui ☐ Non
**Si OUI**	5.b) En général à quelle fréquence ?<br>☐ Au moins une fois par jour ☐ Au moins une fois par semaine ☐ Au moins une fois par mois<br>☐ Moins de une fois par mois	
