# Supplementary material for: Case study of Argus in Togo: An SMS and web-based application to support public health surveillance, results from 2016 to 2019
Source: PLoS One. 2020 Dec 1;15(12):e0243131. doi: 10.1371/journal.pone.0243131 (PMC7707507; doi:10.1371/journal.pone.0243131)
Supplement: Attachment — Submitted filename: response_reviewers.pdf [file pone.0243131.s003.pdf]

Reviewer #1:

The article presents data on the use of an SMS-based surveillance tool from 2016 thru 2019 in two regions within Togo. The goal of the system is to improve reporting of notifiable diseases to the district, regional, and central Ministry of Health in support of IDSR. The SMS tool supports the use of ISDR at all levels of public health. The tool is open source, works with an Android platform, analyzes data using R, and utilizes XML files.

The evaluation of the system is descriptive. The examination did not directly compare paper-based reporting to electronic-supported reporting. Furthermore, the examination did not compare the initial phase with the reinforcement phase. The paper largely presents a collection of data gathered by the team who worked on the implementation. The lack of rigorous methods and limited ability to generalize findings detracts from an interesting report of a novel tool developed for ISDR. While interesting and potentially useful, there are a number of items that need to be addressed before the paper can be further considered for publication.

MAJOR ITEMS

1. A major weakness of the paper is the lack of a clear study design. The paper was submitted as a Research Article. Yet the paper does not appear to be a research study. The paper should follow the STARE-HI guidelines for reporting evaluations of health informatics applications (https://www.imia-medinfo.org/new2/Stare-HI_as_published.pdf). Many aspects of STARE-HI are present in the paper. However, methodological details on study design and evaluation criteria are missing. The paper needs to make it clear in the ABSTRACT and METHODS sections that this is a descriptive study. It further needs to characterize the approach to evaluation. Why was the Evaluation mission so short in duration, even though the paper presents data on a longer time period? With respect to Table 1 in the METHODS, a variety of time periods and measures were used. The table is a handy reference. Yet it is not clear why so many components were used. A clearer vision for the overall evaluation strategy would be helpful. Did the team use a logic model? Were there primary outcomes of interest? Which metrics were most important to the Ministry? How about the health care facilities? Districts? Were the various stakeholders consulted about the evaluation? Who organized and directed the evaluation?

- Thank you for your pertinent comments.
- This is a case study reporting a pilot test of a new IT tool to support public health surveillance in Togo. We updated the paper on several instances to make it clear.
- As mentioned, most of the STARE-HI reporting recommendations were already followed in the paper. We updated the paper to follow as much as possible the remaining ones.
- The implementation and assessment of the pilot test were jointly performed by the Ministry of Health and WHO, this was specified in the paper.
- We clarified the Abstract and Methods to emphasize the descriptive approach and further detail and clarify the methodology used, including the chosen indicators and data collection modalities.
- Below are presented the changes performed to reply to these comments:
    - Title: “*Case study of Argus in Togo: an SMS and web-based application to support public health surveillance, results from 2016 to 2019.*”
    - Abstract:
        - *“This study describes the results of the use of Argus in two regions of Togo.”*

        - *"Argus was used in 148 healthcare facilities from May 2016 to July 2018 expanding to 185 healthcare facilities from July 2018. Data from week 21 of 2016 to week 12 of 2019 was extracted from the Argus database and analysed. An assessment mission took place in August 2016 to collect users' satisfaction, to estimate the concordance of the received data with the collected data, and to estimate the time required to report data with Argus."*
    - Introduction:
        - *"To finalize the development of Argus and assess if it could improve public health surveillance in Togo, Argus had been pilot-tested in two regions of the country since May 2016. In this paper, we describe the results of the Argus pilot test in Togo in terms of data reporting by healthcare facilities, data review by surveillance officers, public health events identified, users' satisfaction and costs."*
    - Methods:
        - *"Argus was implemented in two regions of Togo (Lomé Commune and Savanes) in May 2016 to assess its usefulness in improving public health surveillance."*
        - *"The implementation, operation and assessment of the pilot were conducted under the supervision of the Togolese Ministry of Health with the support of the WHO country office in Togo and WHO Headquarters Lyon office."*
        - *"Outcome measures"*
        - *"An evaluation protocol detailing the indicators to be collected and their collection and analysis modalities was developed by the WHO and agreed upon with the Ministry of Health at the onset of the pilot test. The primary outcomes of interest were the fulfilment of the duties attributed to each level of the surveillance system, i.e. the completeness and timeliness of data reporting and data review. Additional indicators were added to assess: the quality of the reported data (i.e. concordance of the received data with the collected data); the public health events identified (i.e. number of alert thresholds reached and immediate notifications received); the users' satisfaction regarding several characteristics of the system (i.e. appearance, documentation, usefulness, simplicity); the reliability of the system (i.e. percentage of uptime of the Argus web platform); and the costs of implementation and operation. The detailed list of indicators used to assess and describe the Argus pilot test results in Togo is presented in Table 1."*
        - *"To assess data reporting and data review and to describe public health events identified by the system we extracted data from epidemiological week 21 of 2016 to epidemiological week 12 of 2019 from the Argus database. We monitored the percentage of uptime of the Argus web platform through an online monitoring service (https://www.site24x7.com). To describe the costs of the implementation and operation of the system we used the invoices paid for the project and extracted the number of SMS exchanged from the Argus database."*
        - *"One assessment mission took place from the 4th to 12th August 2016 to collect users' satisfaction, to estimate the concordance of the received data with the collected data, and to estimate the time required to report data with Argus. This was done through eight sessions organized with all users of the system".*

        - *“To estimate the concordance of the received data with the collected data, participants were asked to bring the paper forms archived at the healthcare facilities and used to tally the weekly figures. A copy of each form was collected and its data entered twice into a database using the Epidata software.”*
        - *“To estimate the time required to report data with Argus, participants in four of the eight evaluation sessions were provided with a mock paper tallying form and asked to report it using Argus. The amount of time it took each participant to enter the information and send the report was measured and registered by one surveyor.”*
    - Table 1:
        - Title changed to*: “Indicators used to describe the Argus pilot test results”*
        - One additional column was added to specify the type of outcome measure. Rows were sorted to follow the description of outcome measures in the methods, i.e. “Data reporting by healthcare facilities”, “Data review by surveillance officers”, “Public health events identified”, “Users’ satisfaction”, “Costs”.
    - Results:
        - The results sections were reordered accordingly to the outcomes of interests mentioned in the Methods and Table 1, i.e. “Data reporting by healthcare facilities”, “Data review by surveillance officers”, “Public health events identified”, “Users’ satisfaction”, “Costs”.
        - Occurrences of the main events of the pilot study were added to Figures 2 and 4, i.e. “Revision of the reporting list”, “Initial training sessions”, “Assessment mission”, “Reinforcement training sessions”.

2. With respect to development of the Argus tool, who commissioned the tool? WHO? Were the stakeholders like MOH and districts and health care facilities consulted about the development of the tool? Was there any kind of prototype development or usability testing done before implementing in Togo? Were best practices for e-health development utilized? Is the tool part of Togo’s e-Health Strategy as a country? It is not clear why the tool was tested in Togo first rather than another nation.

- As mentioned in the introduction, WHO undertook the development of the tool:
    - *“Dedicated IT tools have already been used in several countries to facilitate reporting and management of public health surveillance data [7,9–13]. In practice, many systems failed to allow remote healthcare facilities to report data due to lack of internet connectivity. Furthermore, we were unable to identify a single tool which would allow each level of the system to review and analyse data from its area in a workflow respecting the IDSR procedures. WHO has therefore developed “Argus”, an IT tool to facilitate public health surveillance for early detection and response of events in compliance with IDSR procedures.”*
- The tool was almost finalized when it was pilot-tested in Togo to 1) ensure the tool was fit for purpose, 2) identify if it could fulfil the needs of the country. We modified the purpose of the study to clarify it:
    - *“To finalize the development of Argus and assess if it could improve public health surveillance in Togo, Argus had been pilot-tested in two regions of the country since May 2016. In this paper, we describe the results of the Argus pilot test in Togo in relation to data reporting by healthcare facilities, data review by*

surveillance officers, public health events identified, users' satisfaction and costs."

- We believe the detailed history of Argus development is out of the scope of this paper, but all details are available in the link mentioned in the paper which provides access to the tool rationale, training and evaluation material, and all the technical details of the tool along with its source code. "*WHO has therefore developed "Argus", an IT tool to facilitate public health surveillance for early detection and response of events in compliance with IDSR procedures (http://www.argus.community/)."*
- By the end of the study, extension of Argus was under consideration by the Ministry of Health and was not yet part of its eHealth strategy. "*Extension of Argus to the whole country is currently under consideration by the Togolese health authorities*."

3. The paper needs a Limitations section where the authors make it clear that improvements in reporting might have been confounded by the fact that the Ministry constrained weekly reporting before the rollout of the tool. Dropping the number of diseases to be reported and reducing the fields to be reported likely facilitated better reporting even without the Argus tool. What variables were dropped from the old paper forms? Why did the ministry constrain reporting for Argus deployment? Did reporting change in the other regions? While a comparison with other regions might not be possible due to data limitations, the fact that close supervision at district, regional, and central levels was associated with better reporting suggests that the Argus tool might have little to do with the outcome. Instead if countries spent more time on these fundamental aspects of IDSR then we might have a more resilient surveillance infrastructure. This should be discussed.

- Dropping the number of diseases to be reported and reducing the fields to be reported was part of the Argus implementation. This has been specified in the methods:
  - *"To make the best use of limited human and financial resources for public health surveillance, the list of events to be weekly reported should be kept to a minimum to ensure that it is manageable by the system and that adequate resources are available to carry out a response. For early detection of outbreaks via weekly aggregated reporting, it was recommended to keep the number of variables to a minimum, ideally reporting only number of cases and deaths, to avoid unnecessary burden on the healthcare facilities and maximize reporting efficiency. In the planning process of Argus, and prior to its implementation, the Ministry of Health revised the list of diseases and conditions to be reported on a weekly basis. "*
- Modifications performed are further detailed in the methods:
  - *"It was reduced from 20 to 13, with acute flaccid paralysis, acute haemorrhagic fever, bloody diarrhoea, cholera, flu syndrome, human anthrax, human rabies, measles, meningitis, neonatal tetanus, other severe diarrhoea, severe acute respiratory syndrome and yellow fever. The variables to be collected were reduced from seventeen variables (mainly number of cases and deaths by age groups) to two: weekly number of cases and weekly number of deaths."*
- We discuss the role of this reduction in the reporting requirements in the discussion:
  - *"We believe the revision by the Ministry of Health of the aggregated data to be weekly reported, with a decrease in the number of diseases and conditions from 20 to 13 and in the number of variables from 17 to 2 was crucial in the adhesion of healthcare facility staff. Due to the study design, the effect of this reduction in the completeness and timeliness of data reporting can't be independently assessed from the use of Argus, but is very likely important."*

- We acknowledged the impossibility to compare our results with the situation before the use of Argus, or in the regions outside the pilot, and we don't speak about "improvements" in reporting due to this fact. Our purpose was to describe the use of Argus for both an extended time-period and area. *"With three years of operation in two regions of the country, our study was able to provide a description of the use of Argus for both an extended time-period and area [15,16]. The lack of monitoring of the completeness and timeliness of weekly reporting by healthcare facilities in the paper-based system prevented temporal comparisons before and after the implementation of Argus and geographical comparisons with regions without Argus. Indeed, only figures of completeness and timeliness of data reporting from the district level were available in the paper-based system.".*
- The role of supervision and the added value of the tool were already discussed*: "The crucial role of supervision at district, regional and central level was highlighted by the relationship between completeness and timeliness of data reporting and data review. It showed that better performance could be achieved when each level took upon its duties in a timely manner. When a drop in the completeness of data review happened at the central and regional level, a drop was also identified in the completeness of data reporting. However, completeness of data reporting remained high in the mainly rural and hard-to-reach region of Savanes. This highlights the added value of data transmission by SMS in remote areas [4]."*
- We fully agree with the reviewer that the most important factors to strengthen public health surveillance systems are not the tools to be used, but rather the fundamental aspects of the system, as mentioned in our concluding paragraph: *"When considering the use of telecommunication and IT tools to improve public health surveillance, countries must also consider other crucial factors such as the integration of data collection and reporting between parallel systems and the limitation in the amount of collected data. Indeed, while the burden put by health information systems on healthcare facility staff is often overwhelming [22–24], minimum data (i.e. weekly number of cases, or alert of an unexpected or serious event) is enough to trigger an investigation."*

4. The number of cases needed for an alert to occur was very small (primarily 1 case). Did an abundance of alerts (N=936 which is approximately 6 per week) contribute to the lackluster use of the tool at the Regional and Central levels? Syndromic surveillance tools have long suffered from "alert fatigue" in the same way as clinical decision support systems. What effect, if any, might this have had on the adoption and use (and enthusiasm) of the Argus tool outside of the health care facilities? This kind of discussion needs to be added to the DISCUSSION section.

- The difference between two types of "alerts" mentioned in the paper was clarified, and the term "alert" is now only used to characterize "alert thresholds"
  - The first type of "alerts" were alert thresholds as defined in the methods:
    - *"Alert thresholds at healthcare facility level were defined for nine diseases and conditions (acute flaccid paralysis: 1, acute haemorrhagic fever: 1, cholera: 1, human anthrax: 1, human rabies: 1, measles: 3, meningitis: 2, neonatal tetanus: 1, yellow fever: 1). If the weekly number of cases reached an alert threshold, a warning message appeared on the Argus Android Client application at the healthcare facility level and the disease or condition was highlighted on the aggregated report displayed on the Argus web platform."*

    - When an alert threshold was reached, no "alert" or message was received by the supervisors, but the disease for which the alert threshold was reached was highlighted on the Argus web platform, we thus don't think it created a "fatigue" as it didn't involve any additional notification or work for surveillance officers (unlike the immediate notification of serious or unexpected events, see below), but rather supported them in identifying quickly diseases for which pre-defined thresholds were reached.
  - The second type of "alert" was the immediate notification by healthcare facility staff of a serious or unexpected event to their supervisor. We added it to the duties at healthcare facility level in the methods section, and we avoided the term of alert for this category in the results section:
    - *"In case of occurrence of a serious or unexpected event, staff at healthcare facilities were required to immediately notify their supervisor, they could do so using Argus. Upon description of the serious or unexpected event on the Argus Android Client application, information was immediately forwarded to the personal mobile phone of pre-identified supervisors by SMS."*
    - *"Sixty-four immediate notification of serious or unexpected public health events were sent by a healthcare facility and forwarded to the personal mobile phone of their supervisor."*

5. The paper makes a point that Argus is open source and designed with interoperability in mind. Yet the paper does not discuss how the tool does or does not integrate with DHIS2, a system used in many countries to support IDSR. Does this tool interface with DHIS2? Are there plans to support use of Argus in conjunction with DHIS2 if some regions in a decentralized country want to use DHIS and others wish to use Argus?

- We have added some information regarding this point. Indeed, DHIS2 was also piloted in the country at the time, but for different purposes, i.e. data warehouse and monthly reporting. As now mentioned in the paper, it was considered to export the data from Argus to DHIS2 on an ongoing manner. Regarding the possibility to interoperate with other tools, the use of XML files is mentioned and the further explanation on the interest of XML for interoperability is available in the provided reference Raja A 2014.
  - *"Through its modular architecture and use of XML files to share information Argus can easily be used with other existing tools [21]. At the Ministry of Health, both the units in charge of the IDSR and of the Health Information Management System were involved in the pilot of Argus. The unit in charge of Health Information Management System was also piloting DHIS2 as a data warehouse and monthly data reporting tool. One option considered by the authorities was to automatize a weekly data export from the Argus instance to the DHIS2 instance."*

MODERATE ITEMS

6. The drop in Week 30 2018 is linked to a system update that included new diseases. Did this correspond with additional training for facilities and ministry staff members? This is not discussed in the DISCUSSION section. It seemed to be a major driver of utilization.

- Yes, as mentioned in the methods, this update corresponds with additional training. *"Prior to the reinforcement phase, the list of diseases and conditions to be reported and the alert thresholds were updated by the Ministry of Health […] Seven training sessions were conducted in July 2018 to update the Argus application on all healthcare facility phones and provide reinforcement training to all staff."*
- We added in the method that this update also corresponds with the designation of a responsible focal point at the central level. We complemented the discussion regarding the impact of the additional training and designation of a responsible focal point at the central level.
  - *"A responsible focal point at the central level was designated to supervise the pilot of Argus during the reinforcement."*
  - *"Reinforcement training and designation of a responsible focal point at the central level at the onset of the reinforcement phase lead to increased performance at all levels."*

7. Some of the detailed data presented in paragraphs in the RESULTS section could become tables that would more succinctly present the results, especially with respect to completeness and timeliness overall and stratified by district, region, and central levels. Furthermore, the differences before and after the reinforcement phase in a table aligned with the initial phase would strengthen comprehension and comparison of these data.

- As proposed, we presented some of the detailed data in the results as a table, displaying side by side results for the overall period, initial phase and reinforcement phase. We agree with the reviewer that it greatly helps comprehension and comparison of data.
  - *See new table 2 in the results section.*

MINOR COMMENTS / SUGGESTIONS

8. The data on phone and computer ownership is not useful as presented. These results are not discussed, nor do these data seem to relate to completeness or timeliness. Consider removing these data.

- We believe information on the level of "technical literacy" of the respondents to the questionnaires is helpful to interpret the results of the questionnaire. This is in line with the recommendations from the STARE-HI: *"When the study measures are related to persons, baseline demographic data and/or (clinical) characteristics of study participants (users, patients, and units) should be given, such as age groups, professions, usage patterns, patients' diagnostic scores, etc. [...]"*
- We would thus like to keep this information in the results section.

9. It would be VERY helpful if the "Evaluation mission" as well as the reinforcement phase were highlighted in Figures 2, 3, and 4. Identifying when the re-training occurred would be helpful to see if this was associated with an uptick in completeness.

- Indeed, this is most helpful. We have added such highlights in figures 2 and 4. As figure 3 is the distribution of the reception time, such highlights cannot be added to this one.

10. The images are quite grainy in Figure 1. As a result it is difficult to read the words in the images. Higher quality images should be provided for publication.

- Higher quality images are available through the link provided on the top right corner of each figure.

Reviewer #2:

I found this paper and the described approach extremely interesting and complete. This kind of mHealth is even more actual in this pandemic phase, during which less progressed countries might have been poorly estimated due to underreporting of events.

What the authors clearly state is the limitation due to lack of engagement. Re-engagement of surveillance officers enabled an increase in completeness of data report . It would be interesting to explain more extensively how re-engagement was performed and which strategies might be implemented to overcome lack of engagement.

- Thank you for the interest in this work.
- We added in the methods section that a responsible focal point at the central level was designated to supervise the pilot during the reinforcement phase:
  - *“A responsible focal point at the central level was designated to supervise the pilot of Argus during the reinforcement phase.”*
- We added actions that may have led to overcome lack of engagement and increase performances:
  - *“Reinforcement training and designation of a responsible focal point at the central level at the onset of the reinforcement phase lead to increased performance at all levels.”*
